# Case Report: A Tale of a Cardiac Mass: Looks Like a Papillary Fibroelastoma, Acts Like a Non-bacterial Thromboendocarditis

**DOI:** 10.3389/fcvm.2021.782926

**Published:** 2021-11-12

**Authors:** Ali Ahmad, Arman Arghami, Edward A. El-Am, Thomas A. Foley, Reto D. Kurmann, Kyle W. Klarich

**Affiliations:** ^1^Department of Cardiovascular Medicine, Mayo Clinic, Rochester, MN, United States; ^2^Department of Internal Medicine, Saint Louis University School of Medicine, Saint Louis, MO, United States; ^3^Department of Cardiovascular Surgery, Mayo Clinic, Rochester, MN, United States; ^4^Department of Medicine, Indiana University School of Medicine, Indianapolis, IN, United States; ^5^Division of Cardiovascular Radiology, Department of Radiology, Mayo Clinic, Rochester, MN, United States; ^6^Heart Center, Kantonsspital Luzern, Lucerne, Switzerland

**Keywords:** papillary fibroelastoma, non-bacterial thromboendocarditis, cardiac mass, marantic endocarditis, case report

## Abstract

**Introduction:** Benign cardiac tumors and tumor like conditions are a heterogeneous collection of mass lesions that vary widely in their characteristics, such as presentation, size, and location. In some instances, these tumors are found incidentally, and therefore a broad differential diagnosis should be considered.

**Case:** An elderly male with significant unintentional weight loss and a high risk for cancer presented with an incidental valvular cardiac mass. The mass was thought to be a non-bacterial thromboendocarditis on initial clinical evaluation. After multiple imaging modalities, the mass was suspected to be a papillary fibroelastoma (PFE), which was resected due to high stroke risk and multiple previous chronic infarcts on brain MRI.

**Conclusion:** This case highlights the need for a comprehensive cardiac evaluation of a valvular tumor to discern the etiology and rule out other underlying pathophysiological processes that may require alternative interventions to cardiac surgery.

## Introduction

Benign cardiac tumors and tumor-like conditionsare a heterogeneous collection of mass lesions that vary widely in location, appearance, size, and presentation. In some instances, the presentations are similar and therefore, a comprehensive assessment would be needed to discern the exact etiology and rule out other etiologies that may require alternative interventions to cardiac surgery.

## Case Report

Written informed consent was obtained from the patient for the publication of any potentially identifiable images or data included in this article.

### History of Presentation

A 73-year-old male smoker presented for the evaluation of a cardiac mass that was diagnosed incidentally on a CT abdomen and thorax. The CT was originally ordered as part of a workup for unintentional 35-lb weight loss over 4 months (240–205 lb). The CT reported an 8-mm polypoid aortic valve along with moderate emphysematous changes in the lungs and several pulmonary nodules (3 mm in size).

He denied any loss of appetite, change in diet, fever, chills, sweating, or other constitutional symptoms. The patient reported adequate functional capacity, exercising twice per week. He also denied any symptoms. The patient provided consent for this case report.

The patient had a history of hereditary hemochromatosis, right carpal tunnel syndrome (treated by surgery), and recently resected early-stage bladder cancer. He also reported a history of severe osteoarthritis treated by surgery and a remote history of basal cell carcinoma on his upper lip that was also resected. Colonoscopy 2 years ago was normal.

### Differential Diagnosis

Papillary fibroelastoma (PFE), healed vegetation, subacute bacterial endocarditis, myxoma, multiple large lambl's excrescences, or non-bacterial thrombotic endocarditis (NBTE) due to occult malignancy or antiphospholipid syndrome.

### Investigations

Laboratory testing revealed mildly elevated hsCRP levels 4.6 ml/L (normal <2.0 mg/L), ESR 38 mm/h, NT-Pro BNP 270 ng/ml (normal <107 pg/ml), and D-dimer 522 ng/ml (normal <500 ng/ml). Other blood studies, such as complete blood count, basic metabolic panel, coagulation studies (INR, PT, aPTT, Fibrinogen, soluble fibrin), and alpha-fetoprotein were normal. Blood cultures were also negative. The antiphospholipid antibody, B-2 glycoprotein, and special coagulation studies were all negative.

**Figure 1:**
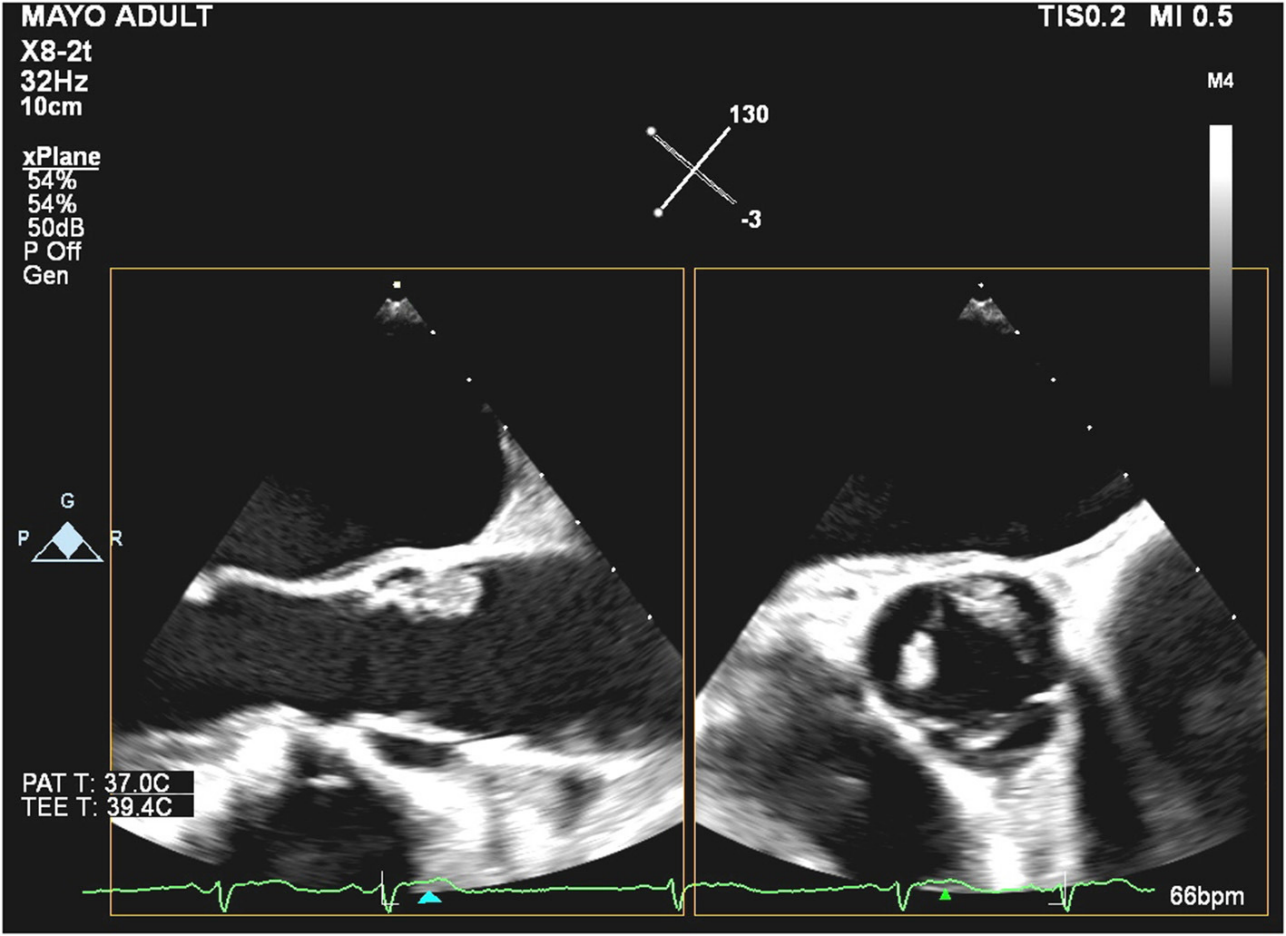
Biplanar transesophageal echocardiography of the aortic valve during systole with the mid-esophageal aortic valve long axis (left) and short-axis (right).

On TEE done at our institution, we identified one large (1.7 × 1.1 cm) mass on the aortic side of the aortic valve, between the non-coronary and left cusp at the base, that is attached by a stalk (Figure 1), likely moving most consistently with the non-coronary cusp (Supplementary Videos 1, 2). In addition, there was a smaller mass at the tip of the left coronary cusp and several mobile echo densities which may represent multiple Lambl's or a sessile type of PFE. The masses on the left-ventricular side of the aortic valve had some features that raised concern for non-bacterial endocarditis.

Brain MRI showed focal encephalomalacia with surrounding FLAIR/T2 hyperintensity that likely reflects gliosis involving the posterior inferior right cerebellar hemisphere, most consistent with effects of chronic infarction, and multifocal chronic-appearing lacunar infarcts involving the cerebellar hemispheres and right parietal lobe.

With the patient's extensive cancer history and to rule out possible lung cancer, an FDG-PET was performed. The study was normal, and no uptake was seen to correlate with the cardiac masses.

**Figure 2:**
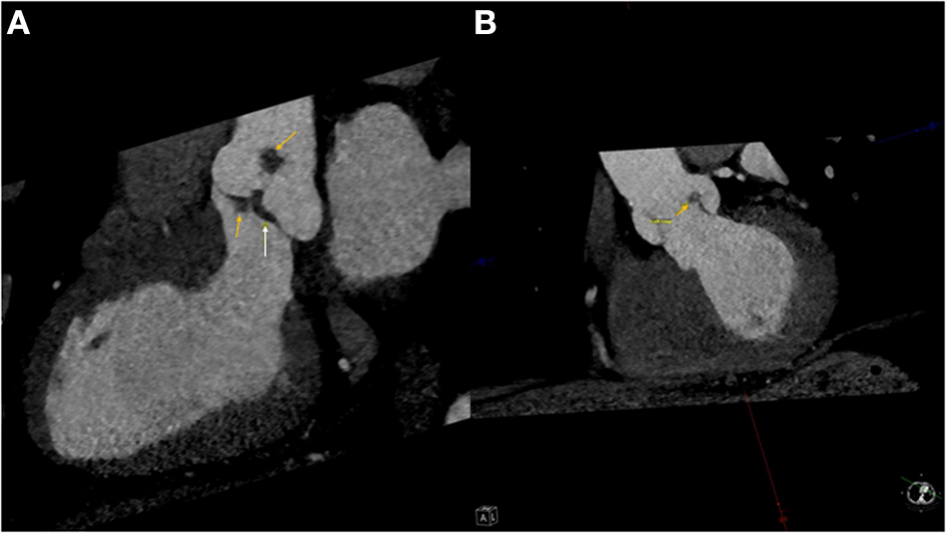
**(A)** Left ventricular outflow tract view of the pre-operative CT angiogram showing the masses (Yellow arrows) and the thickening of the non-coronary cusp of the aortic valve (White arrow). **(B)** An XX view of the aortic valve with indicating the mass on the left cusp of the aortic valve (Yellow arrow).

**Figure 3:**
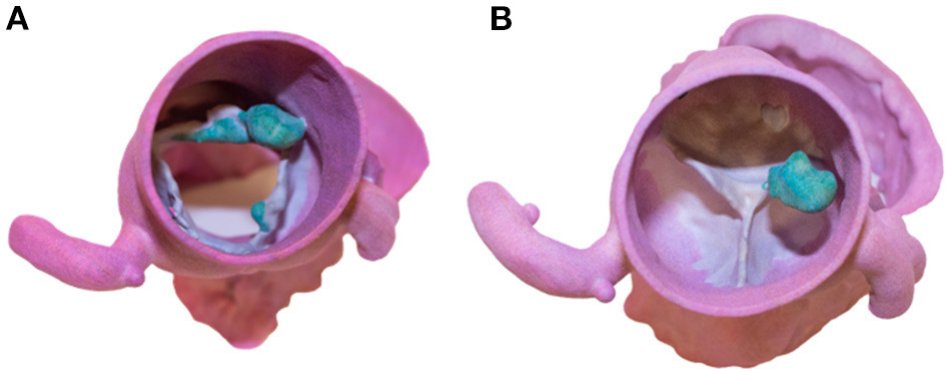
**(A,B)** 3D reconstructed and printed models of the mass.

Pre-operative CT cardiac angiogram with contrast confirmed the presence of the lobular mass and its stalk on the non-coronary cusp, also revealing a more subtle thickening of the left aortic cusp with a more subtle nodule along its ventricular surface, and some thickening more prominent along the ventricular surface of the non-coronary cusp (Figures 2A,B). 3D reconstruction image images were performed before the surgery for clinical and educational purposes and are shown in images Figure 3.

### Management

Given the high suspicion of NBTE and even after the negative workup for cancer, the patient was given 6 weeks of apixaban 5 mg BID to see if the masses would regress, which they did not, based on the follow-up echocardiographic assessment. After discussion with the patient and given the presence of a highly mobile mass on the aortic valve combined with the suspicion of silent emboli on brain MRI, the decision was to excise the mass.

**Figure 4:**
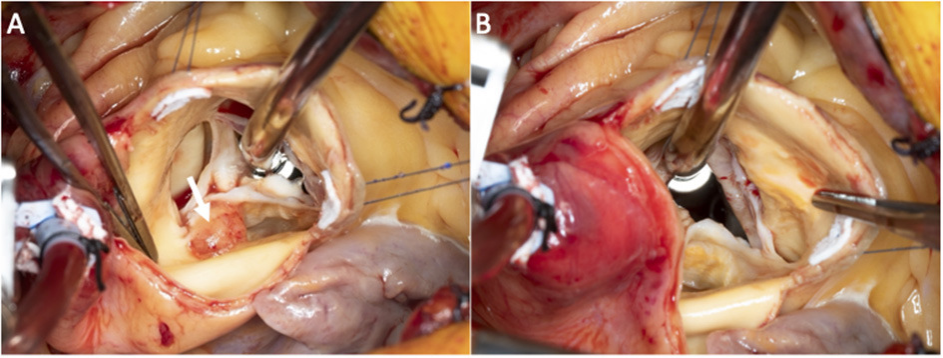
Superior intra-operative images of the aortic valve with the valve closed **(A)** and opened **(B)**.

Pre-bypass TEE confirmed the presence of the mass (no change in size). During surgery, there was one large mass attached to the non-coronary cusp which was about 1.1 cm in diameter. There was a medium size mass attached to the non-coronary cusp which was 2 mm (Figures 4A,B). There were numerous small ≤1 mm frond-like masses attached to the aortic surface of all three cusps. Valve preservation was attained by the meticulous removal of each of the described masses. He had an uneventful recovery, and the dismissal echo showed a well-functioning aortic valve.

**Figure 5:**
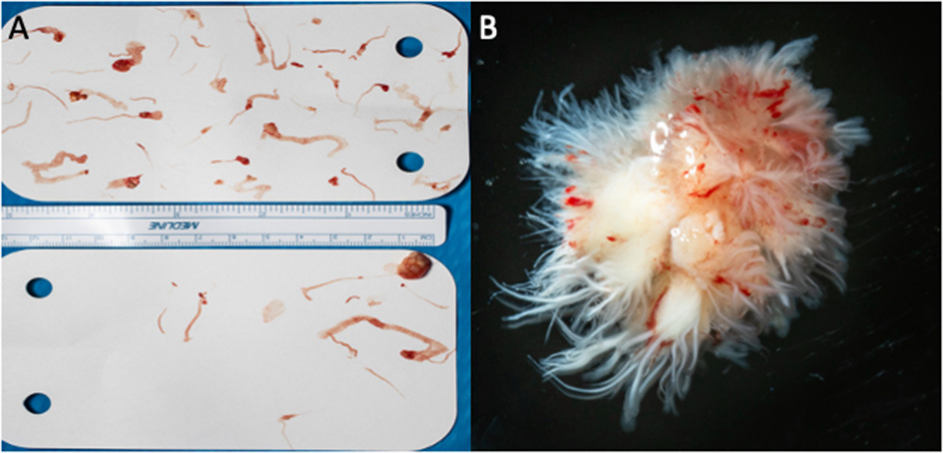
**(A,B)** Gross pathology photographs of the valve.

Gross and microscopic images are shown in Figures 5A,B, which revealed a 1.0 × 0.9 × 0.8 cm papillary mass with numerous (>100) small delicate fronds with multiple fragments of white fibrous and frond-like tissue aggregating to 0.9 × 0.5 × 0.3 cm.

## Discussion

This case makes the important point, that suspected PFE has a differential diagnosis that includes thrombus, and in this age group, NBTE related to cancer is raised as associated with lupus and or antiphospholipid antibody syndrome, and subacute endocarditis. Moreover, it emphasizes how judicious use of imaging can be critical in planning a successful intervention with valve preservation.

The current report describes the case of an elderly male with high cancer risk, due to cancer history and smoking, presenting with weight loss and a cardiac mass. The differential diagnosis for a cardiac mass/vegetation is broad, but only NBTE was previously associated with cancer. However, as we show in this case report, it is important to keep a broad differential until further investigations are done since neither of the differentials is a clinical diagnosis.

Papillary fibroelastoma is being recognized as the most common primary cardiac tumor (1, 2). Edwards et al. analyzed their multi-institutional primary cardiac valve tumors over 58 years and concluded that PFE is the most common primary tumor to arise on cardiac valves (1). This rise in recognition is believed to parallel the increased access to echocardiography in practice and improved imaging capabilities. Papillary fibroelastoma may be a source of systemic embolization or stroke due to the migration of thrombus from the tumor surface or tumor embolization (3–5). Other symptoms include coronary embolism (angina, myocardial infarction, sudden death), pulmonary embolism, and valvular heart disease (3, 4, 6). In a recent study, it was shown that PFE grows slowly in size (7), serial monitoring with echocardiograms may be helpful after anticoagulation to look for regression if NBTE is suspected, where anticoagulation may be a more appropriate therapy than surgery. If serial imaging for the growth of PFE is the goal, then the TEEs should be separated by a fair amount of time due to their slow growth. The decision for excision is recommended for symptomatic patients (3) and consideration of surgical removal of left-sided PFE in an asymptomatic patient with low society of thoracic surgeons' (STS) risk score and, if valvular, there is a great chance of valve preservation. Papillary fibroelastoma do recur at a rate of up to 12% of patients, therefore there may be a role for follow-up echocardiograms after excision (8).

Non-infectious lesions of heart valves, first described as Libman-Sacks, goes by multiple other names, such as NBTE, verrucous endocarditis, or marantic endocarditis (9). It describes a wide array of valvular pathologies due to a non-infectious cause, which could be due to cancer or autoimmune disease (especially systemic lupus erythematosus or antiphospholipid syndrome). Non-bacterial thromboendocarditis is also usually asymptomatic until an embolization occurs. Patients usually present with a cerebrovascular event, although systemic or coronary embolization is also possible (10). Three sets of blood cultures are also needed to rule out subacute bacterial endocarditis. Hypercoagulable studies are also warranted in patients where clinical suspicion is high. PET could also be considered to rule out culture-negative endocarditis by organisms such as *T. whipplei*. In our patient, a PET was also done to exclude underlying malignancy. Treatment of NBTE usually consists of systemic anticoagulation and treating the underlying condition. Excision of the vegetation may have a role in some patients who do not have advanced malignancy.

Cardiac imaging workup for the two pathologies is similar. A TTE is the mainstay imaging modality to describe the vegetation/mass. A TEE should also be considered to discern the details of the vegetations/mass, especially for smaller masses (<5 mm). Papillary fibroelastoma has a typical look of a pedunculated mass with small fronds and a stalk. Non-bacterial thromboendocarditis are usually broad, small (<1 cm), and irregular. In certain cases when the stalk is not visible, or not found, the PFE may look like an NBTE, warranting a TEE or another modality. CT or MRI may also be used to further assess the mass and plan the surgical approach. However, the utility of MRI for cardiac mass work up is not established in literature and echocardiography is still the gold standard due to the high mobility of these masses.

Conclusive diagnosis of either disease is only done through pathological examination of the surgical specimen. A PFE mass is white and fibrous, consisting of endocardial fronds surrounding an avascular collagenous core. An NBTE vegetation consists mainly of immune complexes, mononuclear cells, fibrin, and platelet thrombi.

## Conclusions

This case highlights the need for a comprehensive cardiac evaluation of a valvular tumor to discern the etiology and rule out other underlying pathophysiological processes that may require alternative interventions to cardiac surgery.

## Learning Objectives

- To be able to consider the wide differential diagnosis of valvular masses.
- To learn multiple possible steps in approaching a valvular tumor.

## Data Availability Statement

The original contributions presented in the study are included in the article/Supplementary Material, further inquiries can be directed to the corresponding author/s.

## Ethics Statement

Written informed consent was obtained from the patient for the publication of any potentially identifiable images or data included in this article.

## Author Contributions

AAh, EE-A, and RK, were involved in collecting the data, analyzing them, and writing the report. AAr, TF, and KK were involved in patient care, supervising the case report, and providing a critical review of the written case report. TF was involved in the construction of the 3D model provided in the case. All authors contributed to the writing and revision of the manuscript.

## Conflict of Interest

The authors declare that the research was conducted in the absence of any commercial or financial relationships that could be construed as a potential conflict of interest.

## Publisher's Note

All claims expressed in this article are solely those of the authors and do not necessarily represent those of their affiliated organizations, or those of the publisher, the editors and the reviewers. Any product that may be evaluated in this article, or claim that may be made by its manufacturer, is not guaranteed or endorsed by the publisher.

## Supplementary Material

Supplementary Video 1 Biplanar transesophageal echocardiography of the aortic valve with the mid-esophageal aortic valve long axis (left) and short-axis (right).

Supplementary Video 2 Mid-esophageal long axis view showing the motion of the mass on the left cusp of the aortic valve.

## Abbreviations

AV: aortic valve
NBTE: non-bacterial thromboendocarditis
PFE: papillary fibroelastoma.
