# Supplementary material for: Case Report: A Tale of a Cardiac Mass: Looks Like a Papillary Fibroelastoma, Acts Like a Non-bacterial Thromboendocarditis
Source: Front Cardiovasc Med. 2021 Nov 12;8:782926. doi: 10.3389/fcvm.2021.782926 (PMC8632806; doi:10.3389/fcvm.2021.782926)
Supplement: Supplementary file 3 [file Data_Sheet_1.PDF]

CARE Checklist of information to include when writing a case report

CARE case report guidelines

(cc) BY-NC-ND

Topic	Item	Checklist item description	Reported on Line
Title	1	The diagnosis or intervention of primary focus followed by the words "case report"	Lines 1-2
Key Words	2	2 to 5 key words that identify diagnoses or interventions in this case report, including "case report"	Lines 21
Abstract (no references)	3a	Introduction: What is unique about this case and what does it add to the scientific literature?	Lines 27
	3b	Main symptoms and/or important clinical findings	Lines 32
	3c	The main diagnoses, therapeutic interventions, and outcomes	Lines 32
	3d	Conclusion—What is the main "take-away" lesson(s) from this case?	Lines 37
Introduction	4	One or two paragraphs summarizing why this case is unique (**may include references**)	Lines 51
Patient Information	5a	De-identified patient specific information.	Lines 59
	5b	Primary concerns and symptoms of the patient.	Lines 59
	5c	Medical, family, and psycho-social history including relevant genetic information	Lines 67
	5d	Relevant past interventions with outcomes	
Clinical Findings	6	Describe significant physical examination (PE) and important clinical findings.	
Timeline	7	Historical and current information from this episode of care organized as a timeline	
Diagnostic Assessment	8a	Diagnostic testing (such as PE, laboratory testing, imaging, surveys).	Lines 75
	8b	Diagnostic challenges (such as access to testing, financial, or cultural)	
	8c	Diagnosis (including other diagnoses considered)	Lines 71
	8d	Prognosis (such as staging in oncology) where applicable	
Therapeutic Intervention	9a	Types of therapeutic intervention (such as pharmacologic, surgical, preventive, self-care)	Lines 102
	9b	Administration of therapeutic intervention (such as dosage, strength, duration)	
	9c	Changes in therapeutic intervention (with rationale)	
Follow-up and Outcomes	10a	Clinician and patient-assessed outcomes (if available)	Lines 115
	10b	Important follow-up diagnostic and other test results	Lines 115
	10c	Intervention adherence and tolerability (How was this assessed?)	
	10d	Adverse and unanticipated events	
Discussion	11a	A scientific discussion of the strengths AND limitations associated with this case report	Lines 118-170
	11b	Discussion of the relevant medical literature **with references**.	Lines 118-170
	11c	The scientific rationale for any conclusions (including assessment of possible causes)	
	11d	The primary "take-away" lessons of this case report (without references) in a one paragraph conclusion	Line 172
Patient Perspective	12	The patient should share their perspective in one to two paragraphs on the treatment(s) they received	
Informed Consent	13	Did the patient give informed consent? Please provide if requested	Yes ☐ No ☐
